# Reliability of radiographic measures in infants with clubfoot treated with the Ponseti method

**DOI:** 10.1007/s11832-015-0647-y

**Published:** 2015-03-17

**Authors:** Catherine C. Zimmerman, Blaise A. Nemeth, Kenneth J. Noonan, Timothy P. Vanderbilt, Mark J. Winston, Conor P. O’Halloran, Sarah A. Sund, Scott J. Hetzel, Matthew A. Halanski

**Affiliations:** Department of Orthopedics and Rehabilitation, University of Wisconsin, American Family Children’s Hospital, 1685 Highland Ave., Suite 6170-11D, Madison, WI 53705 USA

**Keywords:** Clubfoot, Radiographic measurements, Reader reliability, Ponseti

## Abstract

**Purpose:**

The aim of this study was two-fold: (1) to determine if radiographic measures can be reliably made in infants being treated with the Ponseti method and (2) to document radiographic changes before and after Achilles tenotomy.

**Methods:**

A retrospective radiographic and chart review was performed on children with clubfoot treated by the Ponseti method at a single institution over a 10-year period. Five independent reviewers measured a series of angles from a lateral forced dorsiflexion radiograph taken prior to and following Achilles tenotomy. These measures were taken in triplicate to determine the intra- and inter-reader reliability of dorsiflexion, tibio-calcaneal, talo-calcaneal, and talo-first metatarsal angles.

**Results:**

Thirty-six subjects (56 feet) were treated with the Ponseti method and met the inclusion criteria. The median (range) age of patients at the time of tenotomy was 52 (34–147) days. The intra-reader reliability [intra-rater correlation coefficient (ICC)] for each of the measured angles pre- and post-tenotomy ranged from 0.933 to 0.995 and 0.864 to 0.995, respectively. Similarly, the inter-reader reliabilities (ICC) ranged from 0.727 for the pre-tenotomy (talo-calcaneal) to 0.950 for the post-tenotomy (talo-first metatarsal) angles. The mean differences between pre- and post-tenotomy radiographs were: dorsiflexion increase of 17°, tibio-calcaneal angle increase of 19°, talo-calcaneal angle increase of 9°, and talo-first metatarsal angle increase of 10° (*p*-value ≤0.001 for all measurements except the talo-first metatarsal angle, with a *p*-value of 0.001).

**Conclusions:**

Reliable radiographic measures can be made from lateral dorsiflexion radiographs of clubfeet treated with the Ponseti method before and after Achilles tenotomy.

## Introduction

Talipes equinovarus (clubfoot) is a complex foot deformity that involves abnormalities of the bones as well as surrounding vasculature and musculature. Historically, the poor understanding of the underlying anatomical deformity has given way to different methods for treating clubfoot and less than satisfactory outcomes [5]. While the etiology of the deformity is still unknown, the improved understanding of the anatomical deformity has led to an improvement in treatment and correction. Multiple studies over the last two decades have demonstrated that the Ponseti method is the preferred method to begin treatment in children with idiopathic as well as syndromic clubfeet. In the former group, the treatment produces a high rate of correction and reduces the need for extensive corrective surgery in the latter group [2, 3, 8, 11].

The initial management of clubfeet by the Ponseti method includes weekly manipulations and casting of the clubfoot, in most cases followed by an Achilles tenotomy. Achilles tenotomies correct the remaining equinus deformity at the end of casting [13]. Correction is then maintained through the use of abduction foot orthoses. Despite the improved understanding and correction of the clubfoot, factors that predict which feet will relapse have not been identified.

Attempts to identify patients or feet “at risk” for a recurrent deformity have been performed. Scoring systems such as those proposed by Pirani and Dimeglio descriptively grade the initial deformity, yet no association between the initial or final Pirani or Dimeglio scores and future surgeries needed to correct residual or resistant deformities has been found [2, 7]. Certain risk factors that increase the likelihood of recurrence have been identified, but these factors are thought to result in recurrence due to decreased bar and shoe wear following casting [6]. While brace wear “non-compliance” has been accepted as a risk factor, clinically, “non-compliance” can be difficult to distinguish from brace wear intolerance—the former being disregarded for the recommended treatment parameters, whereas the latter may be the result of the intrinsic properties of a stiffer foot that makes brace wear uncomfortable. Thus, the identification of objective and reliable parameters that might identify clubfeet at risk of recurrence would be helpful.

Prior to the widespread use of the Ponseti method, radiographic measurements were commonly employed to assess clubfeet and to strategize surgical execution [9, 16, 17]. With the increased popularity of the Ponseti method, radiographic analysis of these feet has become less common. However, the authors believe that radiographic analysis of these feet might provide an objective measure capable of predicting recurrences [4, 15]. Prior to identifying early parameters that may predict recurrences, it is first necessary to demonstrate the reproducibility of these radiographic parameters in very immature feet. The hypothesis of this study was that reproducible radiographic measurements could be made from lateral forced dorsiflexion radiographs pre- and post-Achilles tenotomy in patients treated by the Ponseti method, and that these radiographic measurements would be sensitive enough to determine changes post-tenotomy.

## Materials and methods

The following study was reviewed by the local IRB and (approval/waived).

This study was a retrospective chart review at a single institution of children treated for clubfoot using the Ponseti method over a 10-year period (2001 to 2011) by two physicians. The radiographic images of subjects with the diagnosis of talipes equinovarus (clubfoot) treated by the Ponseti method were reviewed. Subjects at our institution were identified by using ICD 9 codes 736.79, 754.50, 754.51, 754.52, 754.53, 754.59, 754.70, 754.71, and 754.79. See Table 1 for diagnoses associated with ICD 9 codes. From these codes, those that did not have documentation of having talipes equinovarus were excluded. Subjects without pre- and post-tenotomy lateral forced dorsiflexion X-rays of their affected feet were excluded. Our radiographic measures were taken with the foot in maximum dorsiflexion. When performing this radiographic maneuver, the technician used a rigid plastic plate, which was placed under the infant’s foot without attempts to pronate or supinate the foot. At this stage of the treatment, the feet were fully abducted. Using the plate, the foot was maximally dorsiflexed while a lateral radiograph was taken. Different radiographic technicians at two different clinic locations performed the radiographs with no special training, other than verbal instructions and/or feedback, beyond standard technician training. We made no special efforts to provide additional training to the technicians, as we wanted to determine if reliable measures could be obtained between radiographs taken by general radiographic technicians at different institutions. Additional exclusion criteria included prior casting at another institution, previous surgeries to correct foot deformities with no prior casting, other surgeries at the time of their percutaneous Achilles tenotomy after completion of serial casting, and lack of radiographs.

**Table 1:** ICD 9 code and associated diagnosis

ICD 9 code	Associated diagnosis
736.79	Other acquired deformity of ankle and foot
754.50	Congenital talipes varus
754.51	Congenital talipes equinovarus
754.52	Congenital metatarsus primus varus
754.53	Congenital metatarsus varus
754.59	Other congenital varus deformity of feet
754.70	Talipes, unspecified
754.71	Talipes cavus
754.79	Other congenital deformity of feet

Data collected from the charts included demographics, involved foot (bilateral/unilateral), type of clubfoot (idiopathic, teratologic, or complex [14]), initial and final Dimeglio and Pirani scores, age at onset of casting, number of casts needed for initial correction (prior to tenotomy), clinical dorsiflexion, and abduction prior to tenotomy, post-tenotomy. Other medical diagnoses were recorded to identify patients with non-idiopathic (syndromic) clubfeet.

Measurements were performed on de-identified radiographs with the foot in maximum forced dorsiflexion on a lateral film. Measurements included the tibio-calcaneal angle, talo-calcaneal angle, talo-first metatarsal, and an angle of dorsiflexion. The angles were measured by two fellowship-trained pediatric orthopedists and three other physicians at resident or fellowship levels of training. Measurements were standardized using a didactic tutorial on proper measuring techniques utilizing a systematic method, created by the lead author. The readers were required to show proficiency with the electronic measuring tools using the DatCard DICOM Viewer, Pacscube powered by CSView. Consistent and reproducible results on three test subjects defined proficiency. For every image/foot, a reader measured the angle three times. The three angle measurements were used for estimating the intra-reader correlation coefficient. These were then averaged to provide the final angle obtained for that foot/image.

### Radiographic measurements

**Fig. 1:**
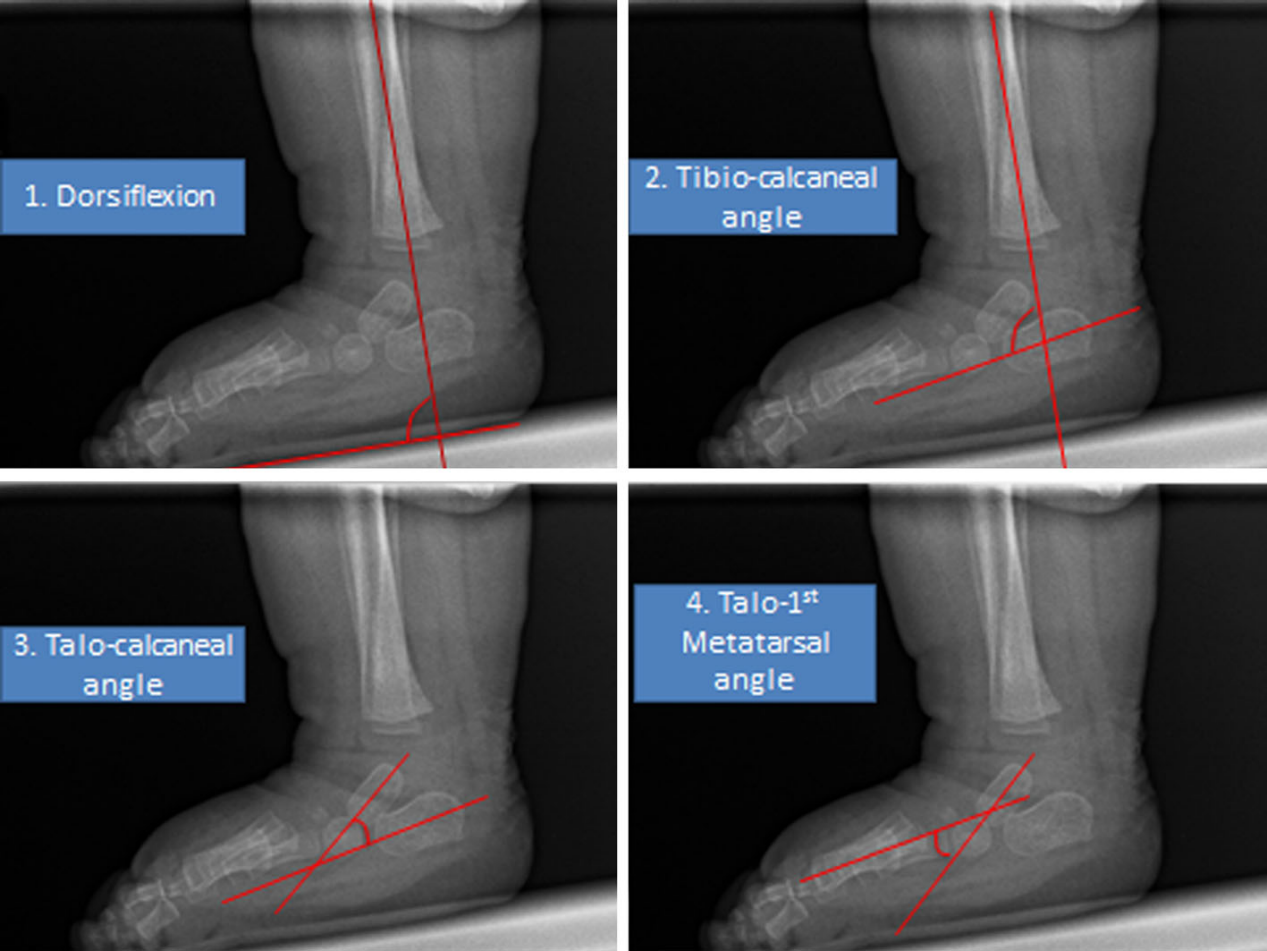
Example of angles measured in this paper

The angle of dorsiflexion was obtained at the intersection of lines drawn along the plantar aspect of the soft tissue shadow of the dorsiflexed foot and the longitudinal axis of the tibia. The lateral tibio-calcaneal angle was measured based on the angle formed by the intersection of a line drawn through the longitudinal axis of the tibia and the line drawn through the long axis of the calcaneus [9, 16]. The tibio-calcaneal angle is a measure or reflection of calcaneal alignment. The talo-calcaneal angle was obtained by drawing a line through the longitudinal axis of the talus and a line through the longitudinal axis of the calcaneus. This angle takes into account three deformities, including hind-foot equinus, varus, and restricted dorsiflexion. The talo-first metatarsal angle measures the alignment of the forefoot with respect to the hind-foot and is formed by the intersection of the longitudinal axis of the talus and that of the first metatarsal [9, 16, 17]. This measure gives information on the presence of a mid-foot breach or rocker-bottom deformity. The position of the talus with regards to the first metatarsal was defined as positive if there was mid-foot breach and negative if there was cavus. An example of the angles measured can be found in Fig. 1.

Intra- and inter-reader reliability was assessed for each of the measurements performed using the intra-rater correlation coefficient (ICC). We used ICC [3,1] for intra-reader reliability and ICC [2,1] for inter-reader reliability [10]. For statistical analysis, we used R version 2.15.

## Results

A total of 206 possible subjects were identified during the study period; 36 subjects (56 feet) met the inclusion criteria. The median age at onset of casting was 16 days (range 4–119 days). The number of casts ranged from 3 to 8 (median 5). The median (range) age of patients at the time of tenotomy was 52 (34–147) days. The median (range) clinical follow-up was 3.34 years (0.13–6.99).

The intra-reader reliability (ICC) for each of the measured angles from the five reviewers is found in Table 2. Similarly, the inter-reader reliabilities (ICC) pre- and post-tenotomy are shown in Table 3. Within the assessment of inter-reader reliability, all of the angles showed strong reliability (ICC > 0.8), except for the right pre-tenotomy talo-calcaneal angle, which showed moderate reliability (ICC 0.6–0.8). In addition, we calculated limits of agreement for each measure based on the Bland–Altman method; these values are listed in Table 3 [1].

**Table 2:** Intra-rater reliability measured with ICC [3,1] (95 % CI)

Reviewer	Measure	Pre-tenotomy	Post-tenotomy
A	Dorsiflexion angle	0.988 (0.981, 0.992)	0.983 (0.973, 0.989)
	Tibio-calcaneal angle	0.962 (0.941, 0.976)	0.975 (0.961, 0.984)
	Talo-calcaneal angle	0.953 (0.927, 0.971)	0.975 (0.962, 0.985)
	Talo-first metatarsal angle	0.983 (0.973, 0.989)	0.989 (0.983, 0.993)
B	Dorsiflexion angle	0.993 (0.989, 0.996)	0.863 (0.797, 0.912)
	Tibio-calcaneal angle	0.95 (0.923, 0.969)	0.992 (0.988, 0.995)
	Talo-calcaneal angle	0.988 (0.982, 0.993)	0.987 (0.98, 0.992)
	Talo-first metatarsal angle	0.995 (0.992, 0.997)	0.995 (0.992, 0.997)
C	Dorsiflexion angle	0.99 (0.985, 0.994)	0.99 (0.985, 0.994)
	Tibio-calcaneal angle	0.975 (0.962, 0.985)	0.98 (0.969, 0.988)
	Talo-calcaneal angle	0.971 (0.955, 0.982)	0.954 (0.93, 0.972)
	Talo-first metatarsal angle	0.977 (0.964, 0.986)	0.977 (0.964, 0.986)
D	Dorsiflexion angle	0.933 (0.899, 0.958)	0.995 (0.993, 0.997)
	Tibio-calcaneal angle	0.992 (0.987, 0.995)	0.994 (0.991, 0.996)
	Talo-calcaneal angle	0.993 (0.989, 0.996)	0.984 (0.975, 0.99)
	Talo-first metatarsal angle	0.994 (0.991, 0.996)	0.995 (0.993, 0.997)
E	Dorsiflexion angle	0.99 (0.984, 0.994)	0.913 (0.868, 0.945)
	Tibio-calcaneal angle	0.986 (0.979, 0.992)	0.988 (0.981, 0.992)
	Talo-calcaneal angle	0.985 (0.977, 0.991)	0.985 (0.977, 0.991)
	Talo-first metatarsal angle	0.989 (0.983, 0.993)	0.99 (0.985, 0.994)

**Table 3:** Inter-rater reliability measured with ICC [2,1] (95 % CI)

Measure	Time	ICC (95 % CI)	Limit of agreement
Dorsiflexion	Prior to casting	0.871 (0.815, 0.915)	13.9
	Post cast removal	0.933 (0.903, 0.957)	9.2
Tibio-calcaneal	Prior to casting	0.897 (0.852, 0.932)	13.9
	Post cast removal	0.910 (0.870, 0.941)	13.2
Talo-calcaneal	Prior to casting	0.727 (0.630, 0.814)	22.6
	Post cast removal	0.925 (0.889, 0.952)	12.3
Talo-first metatarsal	Prior to casting	0.934 (0.903, 0.958)	15.6
	Post cast removal	0.950 (0.928, 0.968)	14.8

The mean (95 % CI) angular measurement changes (pre-tenotomy to post-tenotomy) for each of the angles measured is found in Table 4. Dorsiflexion increased by an average of 17° (13°–20°), the tibio-calcaneal angle increased 19° (15°–23°), the talo-calcaneal angle increased 9° (6°–13°), and the talo-first metatarsal angle increased 10° (4°–16°) (*p*-value ≤0.001 for all measurements except the talo-first metatarsal angle, which had a *p*-value of 0.001).

**Table 4:** Mean angular changes from pre-tenotomy to post-tenotomy radiographs

Measure	Pre-tenotomy	Post-tenotomy	Post–pre	*p*-Value
Dorsiflexion angle	9 (13)	26 (13)	17 (13–20)	<0.001
Tibio-calcaneal angle	1 (15)	20 (16)	19 (15–23)	<0.001
Talo-calcaneal angle	30 (14)	39 (15)	9 (6–13)	<0.001
Talo-first metatarsal angle	20 (21)	30 (22)	10 (4–16)	0.001

Measured as mean (SD) and mean (95 % CI)

## Discussion

Ponseti felt that radiographs were not needed to quantify clubfoot deformity and preferred to depend on physical exam to guide treatment [13]. Others have suggested that pre-treatment radiographs would be difficult to measure in these small patients. In our practice, we have chosen to make our radiographic measures after the bulk of the forefoot and mid-foot deformity was corrected with the Ponseti method and prior to and after the final component of equinus correction with heel-cord release. We have felt that measures would be easier and more reproducible in these feet that are more developed and we would be able to standardize foot position with a forced dorsiflexion lateral.

In this study, we demonstrate that reliable radiographic measurements can be made using lateral forced dorsiflexion foot radiographs taken in infants with clubfeet. Our results are similar to the previous report by Radler et al. [15], yet we also noted where some differences were also found. The lateral tibio-calcaneal angle changes were similar, 19.1° in our study compared to 16.9° in the previous report; however, the lateral talo-calcaneal angle changed by 9.3° in our study compared with only 1.4° in their report. While the clinical significance of this difference is uncertain, the overall trend of a greater change in the lateral tibio-calcaneal angle than the lateral talo-calcaneal angle has been reported by others [4]. The limit of agreement for each measure at each time point showed that the majority of the measurements have limits of agreement near 12°–15°. This indicates that 95 % of the differences between raters will lie between ± 12°–15°, which gives us a sense of what a meaningful difference between two subjects would be.

While this report is similar to the study by Radler et al. [15], significant differences exist. The present study was performed using radiographs from a single institution measured by multiple readers, as opposed to radiographs taken from different institutions, where techniques may vary. Furthermore, the present study is the largest series (by number of subjects) to date to demonstrate the reliability of these radiographic measurements. Likewise, this study utilized more readers with differing levels of expertise than any of the previous reports [4, 15]. Finally, we used radiographs from patients with clubfeet that were syndromic as well as from patients with idiopathic clubfeet. The inclusion of both groups allowed for a large enough cohort to demonstrate acceptable intra- and inter-reader reliability, but analysis of separate phenotypic subgroups was not possible and may account for some of the differences between this manuscript and that of Radler et al. [15]. We do not believe that the inclusion of patients with non-idiopathic clubfeet detracts from this study’s primary goal of documenting the reliability of measurements taken in clubfeet partially treated with the Ponseti method and just prior to tenotomy.

The forced dorsiflexion lateral radiograph is easy to obtain in the clinical setting and requires little training of the radiographic technician, other than verbal instruction. At our institution, there are no dedicated technologists for the pediatric orthopedic clinic, as all radiographic technicians at the hospital rotate on a weekly basis. While the argument could be made that having multiple technicians without additional formalized training performing these radiographs might lead to a variation in technique, the authors believe that this potential variation strengthens the applicability of our findings to other institutions and clinical settings. The primary weaknesses of this study are related to its retrospective nature. As with any retrospective review, the information gathered was only as good and complete as the documentation found in the charts. Many subjects had to be excluded due to a lack of documentation, lack of radiographic imaging, or unobtainable images.

Taken with the study by Radler et al. [15], our work demonstrates the high reproducibility of measurements that can be made from lateral dorsiflexed foot radiographs in infants with clubfeet. This high level of reliability validates the use of such measurements for future studies. These findings will allow others to use such measurements to look for early objective radiographic measures that may be predictive of later clubfoot recurrences. Since the end of this study, the authors have used these data on an expanded clubfoot population to identify at least one objective radiographic measurement(s) that appears predictive; these data have recently been published [12]. In summary, reproducible radiographic measurements can be made using forced lateral radiographs in infants with clubfeet by readers of varying levels of experience. Correlating these measurements with later clinical outcomes may provide clinicians with an early objective manner in which to identify feet at risk of recurrence.
